# Unilateral Isolated Congenital Ectopic Pupil (Corectopia) in a Four-Year-Old Child: Case Report and Literature Review

**DOI:** 10.7759/cureus.45090

**Published:** 2023-09-12

**Authors:** Walid M Abdalla, Nabeela Sharef, Rawan Alhalabi

**Affiliations:** 1 Ophthalmology, Orbit Eye Center, Dubai, ARE; 2 Optometry, Orbit Eye Center, Dubai, ARE; 3 Pediatrics, American Hospital Dubai, Dubai, ARE

**Keywords:** tractional corectopia, congenital eye diseases, corectopia, pupil membrane, ectopic pupil

## Abstract

Unilateral corectopia is an exceedingly rare congenital defect where the pupil is displaced from its central position. Usually, it presents with normal visual aperture or associated with other diseases. We describe the first reported case of a left ectopic pupil in a healthy four-year-old boy with normal lens structure and total lack of visual aperture. Reporting such instances contributes to the understanding of this condition and guide future research endeavors. Further studies are needed to reveal the underlying pathophysiology, refine treatment approaches, and assess long-term outcomes.

## Introduction

Congenital defects of the pupil are uncommon. Corectopia (ectopic pupil) is a congenital pupil abnormality characterized by bilateral pupil displacement from their central position [1,2]. Generally, it presents as Ectopia Lentis et Pupillae (ELeP), a rare congenital condition where the pupil and lens are displaced in opposite directions. ELeP is usually associated with other systematic diseases [1,3,4]. Here, we report an exceedingly rare instance of an isolated unilateral corectopia in a child while no other abnormalities were detected.

## Case presentation

A four-year-old boy presented to our clinic with a complaint of left eye deviation persisting for six months. There was no history of trauma, and the child had no other relevant medical conditions. Family history was irrelevant.

**Figure 1:**
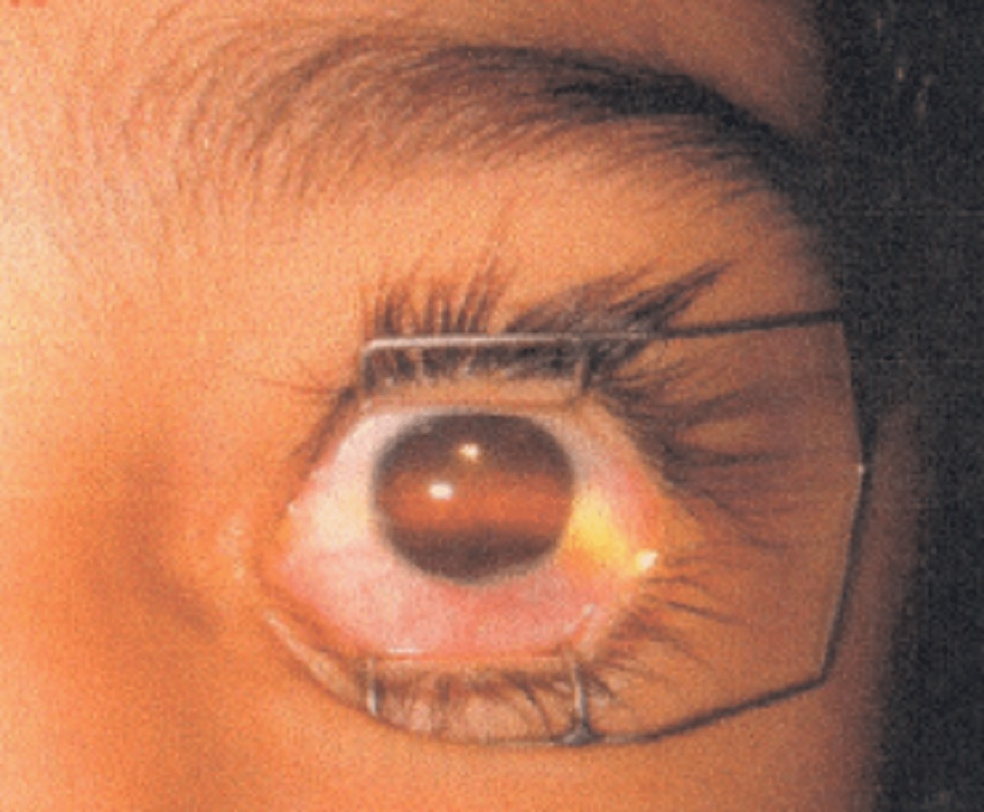
Slit lamp examination revealed the absence of a pupil in the left eye

**Figure 2:**
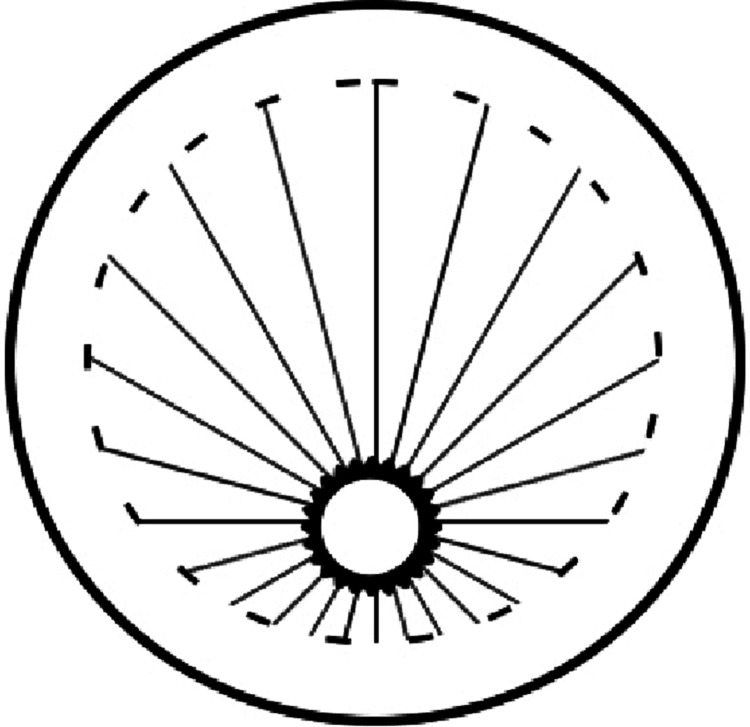
An approximate illustration of the left eye pupil position

On examination, he exhibited 20/20 visual acuity in the right eye, while the left eye only demonstrated the ability to perceive hand movements. The patient was able to track light in all directions of gaze. A slit lamp examination revealed the absence of a pupil in the left eye (Figure 1). Also, Figure 2 shows an approximate illustration of the pupil.

**Figure 3:**
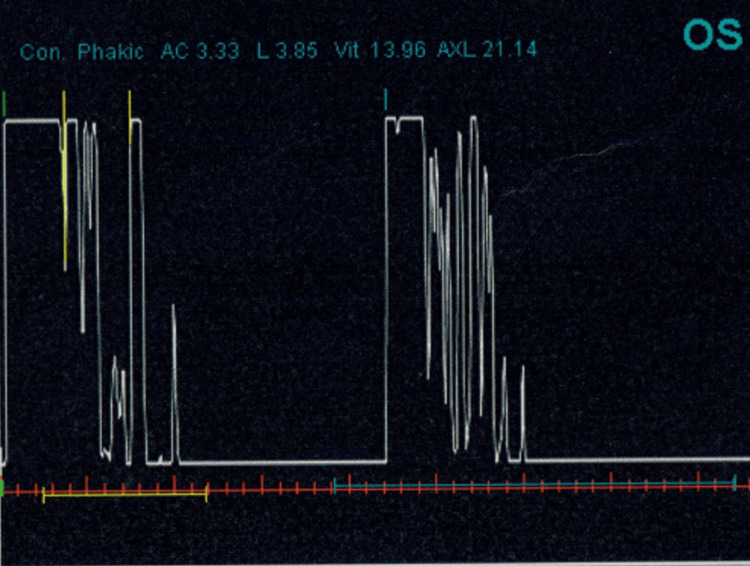
Ultrasound A-scan demonstrating normal globe structure in the left eye

**Figure 4:**
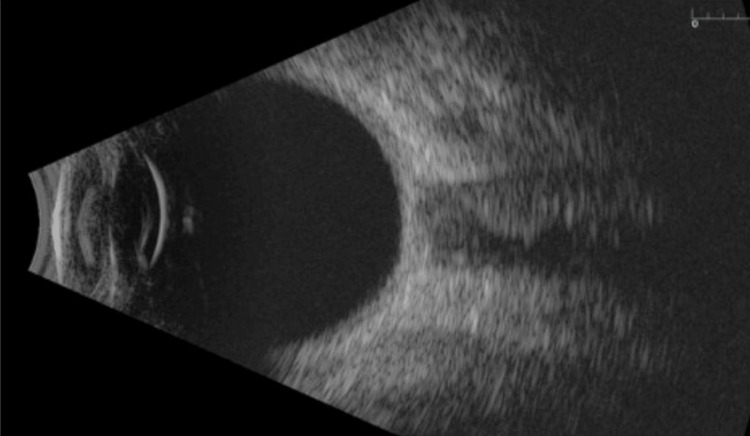
Ultrasound B-scan demonstrating normal globe structure in the left eye

Under laryngeal mask anesthesia, the pupil was observed to be located at the 6 o'clock position with no visible aperture. Ultrasonography (A and B scan) demonstrated a normal globe structure as shown in Figures 3, 4. The iris appeared otherwise normal. Aside from the ectopic pupil, no other abnormalities were detected. Both eyes exhibited similar characteristics behind the pupil, with no signs of cataract formation, normal intraocular pressure, intact retinas, and an average axial length.

The parents were informed about the potential of a poor visual outcome associated with any surgical intervention, particularly pupiloplasty, due to the anticipated development of sensory-deprived amblyopia. However, the patient did not follow up thereafter.

## Discussion

Early reports of unilateral isolated ectopic pupil are inadequate. To our knowledge, only seven patients were reported to have isolated unilateral tractional corectopia since 1930 [1,2,5,6]. This case exhibits distinct characteristics in terms of age of presentation and the visual aperture. These variations underscore the heterogeneity of this condition and the potential influence of various underlying factors. Table 1 shows the comparison between our case and the reported cases in the literature. 

**Table 1:** Reported cases of isolated unilateral tractional corectopia compared to our case

Paper	Age of presentation	Manifestations	Management	Outcomes
Kumar et al. [1]	Almost immediately after birth	* Unilateral (right) misshapen eccentric pupil	* Occlusion therapy was commenced; the eye became visually unresponsive * Central pupil was created by small multiple iris sphincter incisions	The eye settled well without complications. The vision had improved to 6/24.
		* Fibrous band extending from the pupil margin to the endothelial surface of the peripheral supero-nasal cornea		
		* A pupil opening was present with normal red reflex and limited view of the ocular fundus		
Mathur [2]	30 years old	* The left eye pupil was displaced medially and elliptical in shape	The right eye needed - 1-75 D sph.,- 2.5 D cyl., axis 110°.	The visual acuity in the right eye could be improved to 6/6-2. The left eye could not be improved.
		* Reactions to light were brisk except from the medial side		
		* The visual acuity was 6/24 in the right eye and 2/60 in the left		
Rajasekharan et al. [5]	50 years old	* The left eye showed a small eccentrically placed pupil with reduced papillary diameter	Not needed	N\A
		* His visual acuity, visual fields, and optic fundi were normal		
Atkinson et al. [6]	Almost immediately after birth	* Isolated unilateral corectopia in 4 infants, resulting from a white band that extended from the pupil margin to insert in a circumferential condensation of tissue on the endothelial surface of the peripheral cornea that superficially resembled an incomplete posterior embryotoxon	Two who developed shallow anterior chambers were treated surgically, one with an Nd:YAG laser and the other with incisional surgery. The third was treated with medical mydriasis.	All four children have had favorable visual outcomes to date.
		* Amblyopia was not present in any of the patients		
This case	4 years old	* The left eye only demonstrated the ability to perceive hand movements	N\A	N\A
		* Pupil was observed to be located at the 6 o'clock position with no visible aperture with no fibrous membrane noted.		

While the exact prevalence remains uncertain due to the scarcity of cases, its uniqueness and clinical implications cannot be understated. Some have suggested genetic defects leading to abnormal membranes formation arranged in radiating folds dragging the pupil, remnants of anterior tunica vasculosa lentis membrane, and the potential of fetal iritis [3,4,6,7]. These hypotheses, while theoretical, offer perceptions into possible mechanisms that necessitate further investigation.

Few cases were reported to be associated with syndromes like ELeP, persistent pupillary membrane, Axenfeld-Reiger anomaly, Rieger syndrome, or Myhre syndrome [1,3,4,8-10]. However, our case did not demonstrate any other manifestations suggestive of syndromes.

Limited reports suggested satisfactory results following restricted interventions. Atkinson et al. described three patients with corectopia. The treatment plan included an Nd:YAG laser for one baby, and incisional surgery for another one. The third was treated with medical mydriasis. All children have had favorable visual outcomes [6]. Griener al. and Yang et al. supported the use of Nd:YAG laser for managing congenital persistent pupillary membrane [10,11]. The authors emphasized that provided the physician is skillful, with a proper laser energy is used, no significant sequalae will occur [11]. We hypothesize that the surgical methods, including Nd:YAG laser, might have been helpful in our case if a radiating membrane or the visual aperture were noted, and procedure had been performed almost immediately after birth.

In the light of a poor visual outcome associated with any surgical intervention, particularly pupiloplasty, due to the anticipated development of sensory-deprived amblyopia, we assume that performing surgical intervention to this case will not be determinantal.

Although unilateral corectopia is a rare occurrence, its clinical significance should not be ignored. This case highlights the importance of reporting such instances to contribute to the understanding of this condition and guide future research endeavors. Further studies are needed to reveal the underlying pathophysiology, refine treatment approaches, and assess long-term outcomes.

## Conclusions

To conclude, while the clinical picture and the genetic features of ELeP and bilateral corectopia are recognized in the literature, isolated unilateral corectopia has received minute concern. Restricted papers proposed reasonable results with Nd:YAG laser. However, we hypothesise that the surgical methods might be helpful if a radiating membrane or the visual aperture were noted. Despite concerns about surgical interventions and sensory-deprived amblyopia, the need to contribute to the knowledge pool, refine treatments, uncover pathophysiology, and assess outcomes remains pivotal. This report underscores the significance of investigating isolated unilateral corectopia, supplementing existing literature on more recognized pupil abnormalities, and guiding future research directions.
